# Characterising Plant Deubiquitinases with *in vitro* Activity-based Labelling and Ubiquitin Chain Disassembly Assays

**DOI:** 10.21769/BioProtoc.4015

**Published:** 2021-05-05

**Authors:** Michael J. Skelly, Steven H. Spoel

**Affiliations:** Institute of Molecular Plant Sciences, School of Biological Sciences, University of Edinburgh, Edinburgh, United Kingdom

**Keywords:** Ubiquitin, Deubiquitinase, Ubiquitin vinyl sulfone, Proteasome, Cell signaling, Proteostasis

## Abstract

Post-translational modification of proteins by ubiquitin is an essential cellular signaling mechanism in all eukaryotes. Ubiquitin is removed from target proteins by a wide range of deubiquitinase (DUB) enzymes with different activities and substrate specificities. Understanding how DUBs function *in vitro* is a vital first step to uncovering their cellular roles. Here, we provide protocols for the rapid analysis of DUB activity *in vitro* by activity-based labelling with the suicide probe, HA-ubiquitin vinyl sulfone (HA-UbVS), and ubiquitin chain disassembly assays. We have previously used these methods to analyse the activity of the *Arabidopsis thaliana* DUB, UBP6, but in principle, these protocols are applicable to any DUB of interest.

## Background

Regulation of cellular protein homeostasis (proteostasis), the intricate balance between protein synthesis, modification, and degradation, is essential for the survival of all organisms (Balch *et al.*, 2008). Proteostasis is maintained by a diverse range of post-translational modifications (PTMs) that increase the size and functionality of proteomes (Skelly *et al.*, 2016). Ubiquitination, the covalent attachment of the small regulatory protein ubiquitin to target proteins, is a prevalent PTM involved in numerous cellular processes in eukaryotes. Ubiquitin is conjugated predominantly to lysine residues of target substrates via its C-terminus, which requires the sequential actions of three types of enzyme; E1 ubiquitin-activating enzymes, E2 ubiquitin-conjugating enzymes, and E3 ubiquitin ligases. Ubiquitin itself contains seven lysine residues that can also be modified, facilitating formation of polyubiquitin chains of various linkage types. Different ubiquitin chain topologies have specific signalling roles and are associated with different cellular processes. For example, K48-linked chains generally target proteins for degradation by the proteasome, a large multi-protein complex that is responsible for the majority of proteolytic activity in eukaryotic cells. However, ubiquitin modifications can also be reversed by the action of a wide range of deubiquitinase (DUB) enzymes with diverse specificities for different ubiquitin linkage types (Mevissen and Komander, 2017). Since deubiquitination determines the stability, activity, or function of proteins, entire proteomes can be shaped by the activities of specific DUBs. Many human diseases, including cancers, are associated with dysregulated DUB activity, demonstrating the physiological and pathological importance of these enzymes (Harrigan *et al.*, 2018; Clague *et al.*, 2019).

To understand DUB function, their activity can be analysed by various *in vitro* methods to determine substrate specificity and regulation of activity (Cho *et al.*, 2020). These methods include activity-based protein profiling using synthetic ubiquitin suicide probes that irreversibly label DUB active sites (Borodovsky *et al.*, 2002; Gong *et al.*, 2018; Schauer *et al.*, 2020), as well as assays in which purified ubiquitin chains are incubated with DUBs and chain disassembly is monitored by western blotting or gel staining. Recently, we used these methods to uncover the signalling roles of the *Arabidopsis thaliana* proteasome-associated deubiquitinases, UBP6 and UBP7, in plant immunity (Skelly *et al.*, 2019). Here, we describe protocols for analysing recombinant UBP6 activity by HA-UbVS labelling and *in vitro* deubiquitination assays. Importantly, these methods can be applied to any DUB of interest and in the case of HA-UbVS, labelling can also be applied to protein extracts from any organism to uncover proteome-wide DUB activity. Characterising the activity and regulation of DUBs is vital to understand how these enzymes act *in vivo* and will advance the fields of ubiquitin signalling and proteostasis across eukaryotes.

## Materials and Reagents

1. HA-ubiquitin-vinyl sulfone (Boston Biochem, catalog number: U-212)
2. 26S proteasome (Ub-VS treated) (Ubiquigent, catalog number: 65-1020-010)
3. Recombinant purified His_6_-T7-UBP6 (Skelly *et al.*, 2019) or other DUB of interest
4. Ubiquitin subtrates for deubiquitination assays:
  Poly-ubiquitin (Ub3-7) K48-linked (Boston Biochem, catalog number: UC-220)
  Poly-ubiquitin (Ub3-7) K63-linked (Boston Biochem, catalog number: UC-320)
  Di-ubiquitin K48-linked (Boston Biochem, catalog number: UC-200B)
  Di-ubiquitin K63-linked (Boston Biochem, catalog number: UC-300B)
5. UltraPure Tris (Invitrogen, catalog number: 15504-020)
6. MgCl_2 _hexahydrate (Sigma-Aldrich, catalog number: M2670)
7. Adenosine triphosphate (ATP) (Sigma-Aldrich, catalog number: A26209)
8. Dithiothreitol (DTT) (Sigma-Aldrich, catalog number: 1114740005)
9. Glycerol (Sigma-Aldrich, catalog number: G5516)
10. Sodium dodecyl sulfate (SDS) (Sigma-Aldrich, catalog number: L3771)
11. Bromophenol blue (Sigma-Aldrich, catalog number: B8026)
12. Anti-HA antibody (ThermoFisher, catalog number: 26183)
13. Anti-ubiquitin antibody (Santa Cruz Biotechnology, catalog number: sc-8017)
14. Antibody against the DUB being analysed, *e.g*., anti-T7 for His_6_-T7-UBP6 (Millipore, catalog number: 69522)
15. Double-distilled H_2_O (ddH_2_O)
16. 15% SDS-PAGE gel (*e.g.*, compatible with Bio-Rad Mini-PROTEAN system)
17. SuperSignal West Pico PLUS Chemiluminescent Substrate (ThermoFisher, catalog number: 34577)
18. 10× DUB labelling buffer (store at -20°C) (see Recipes)
19. 10× deubiquitination buffer (store at -20°C) (see Recipes)
20. 2× SDS-PAGE loading buffer (see Recipes)
21. DUBs, proteasomes, HA-UbVS, and ubiquitin substrates (see Recipes)

## Equipment

1. 1.5 ml microcentrifuge tubes (*e.g.*, Starlab, catalog number: S1615-5500)
2. Pipettes and tips (*e.g.*, Gilson Pipettman, various volumes)
3. Heat block (*e.g.*, ThermoFisher, catalog number: 88870004)
4. Protein gel electrophoresis and western blotting apparatus (*e.g.*, Bio-Rad Mini-PROTEAN system)

## Procedure

- HA-UbVS labelling
  1. Set up 10-μl reactions including the following: 1 μl 10× DUB labelling buffer, 5 μl 700 nM DUB, 1.25 μl 80 nM 26S proteasome (if required), 1 μl 7 μM HA-UbVS, and 1.75 μl ddH_2_O. Control samples should be included in which components are omitted and replaced with ddH_2_O.
  2. Incubate for 30 min at room temperature or in a heat block at 22°C.
  3. Add 10 μl 2× SDS-PAGE loading buffer to each sample.
  4. Incubate for 10 min in a heat block at 70°C.
  5. Perform SDS-PAGE with a 15% gel and western blotting using standard methods with:
    - Anti-HA antibodies (at 1:5000 dilution) to detect free HA-UbVS (~9.8 kDa) and HA-UbVS-labelled DUB (Figure 1).
    - Antibodies (at antibody-specific dilutions) against the appropriate DUB or epitope tag to detect both unlabelled and labelled DUB. Any standard imaging system can be used depending on the secondary antibodies (*e.g.*, film or CCD imaging system for chemiluminescence).
    **Figure 1.**
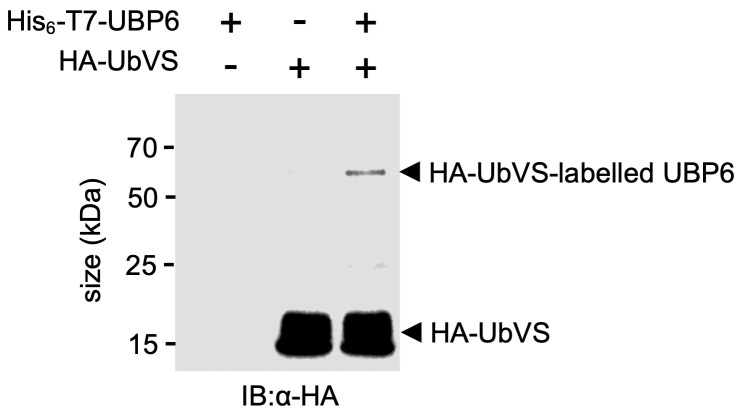
HA-UbVS labelling of recombinant purified His_6_-T7-UBP6. A typical western blot showing free and HA-UbVS-labelled recombinant His_6_-T7-UBP6. Adapted from Figure 6B ofSkelly *et al.* (2019).
- *In vitro* deubiquitination assays
  1. Set up 10 μl reactions including the following: 1 μl 10× deubiquitination buffer, 1 μl 200 nM DUB, 1 μl 12.5 nM 26S proteasome (if required), 1 μl 4 μM ubiquitin substrate, and 6 μl ddH_2_O. Control samples should be included in which components are omitted and replaced with ddH_2_O.
  2. Incubate in a heat block at 30°C for the desired time [2-16 h for His_6_-T7-UBP6 (Skelly *et al.*, 2019), but dependent on the DUB and substrate].
  3. Add 10 μl 2× SDS-PAGE loading buffer to each sample.
  4. Incubate for 10 min in a heat block at 70°C.
  5. Perform SDS-PAGE with a 15% gel and western blotting using standard methods with anti-ubiquitin antibodies to detect the original ubiquitin chain substrate and observe any DUB-mediated chain disassembly as lower-order ubiquitin species. Any standard imaging system can be used depending on the secondary antibodies (*e.g.*, film or CCD imaging system for chemiluminescence).

## Recipes

1. 10× DUB labelling buffer (store at -20°C)
  500 mM Tris pH 7.4
50 mM MgCl_2_
  10 mM DTT
  10 mM ATP
2. 10× deubiquitination buffer (store at -20°C)
  500 mM Tris pH 7.4
50 mM MgCl_2_
  10 mM DTT
  50 mM ATP
3. 2× SDS-PAGE loading buffer (store at room temperature, add DTT fresh before use)
  20% glycerol
  120 mM Tris pH 6.8
  4% SDS
  0.02% Bromophenol Blue
  100 mM DTT
4. DUBs, proteasomes, HA-UbVS, and ubiquitin substrates
Diluted in ddH_2_O from stocks to the required concentrations described above
